# Formulation and Optimization of Nanospanlastics for Improving the Bioavailability of Green Tea Epigallocatechin Gallate

**DOI:** 10.3390/ph14010068

**Published:** 2021-01-15

**Authors:** Eman A. Mazyed, Doaa A. Helal, Mahmoud M. Elkhoudary, Ahmed G. Abd Elhameed, Mohamed Yasser

**Affiliations:** 1 Department of Pharmaceutical Technology, Faculty of Pharmacy, Kaferelsheikh University, Kaferelsheikh 33516, Egypt; 2 Department of Pharmaceutics, Faculty of Pharmacy, Fayoum University, Fayoum 63514, Egypt; das12@fayoum.edu.eg; 3 Department of Pharmaceutical Chemistry, Faculty of Pharmacy, Horus University, New Damietta 34518, Egypt; melkhodary@horus.edu.eg; 4 Department of Pharmacology and Toxicology, Faculty of Pharmacy, Mansoura University, Mansoura 35516, Egypt; Ahmed_gamal_helal@mans.edu.eg; 5 Department of Pharmacology, Faculty of Pharmacy, Horus University, New Damietta 34518, Egypt; 6 Department of Pharmaceutical Technology, Faculty of Pharmacy, Horus University, New Damietta 34518, Egypt; mhamdy@horus.edu.eg

**Keywords:** EGCG, spanlastics, edge activator, bioavailability, optimization

## Abstract

The present study aimed to investigate the potential of nanospanlastics for boosting the bioavailability of epigallocatechin gallate (EGCG). EGCG has valuable effects like anti-inflammation, anti-oxidation, and anti-tumorigenesis. Unfortunately, it has a low oral bioavailability due to its limited permeation and poor stability. To overcome these pitfalls, EGCG was fabricated as a nanospanlastic. Nanospanlastics are flexible nanovesicles that are composed of surfactants and edge activators (EAs). EAs improve the deformability of spanlastics by acting as a destabilizing factor of their vesicular membranes. EGCG-loaded spanlastics were prepared by an ethanol injection method, according to 2^3^ factorial design, to explore the impact of different independent variables on entrapment efficiency (EE%), % drug released after 12 h (Q_12h_), and particle size (PS). In vitro characterization, ex vivo intestinal permeation test, and pharmacokinetic study of the optimized formula were performed. A newly developed RP-HPLC technique was adopted for the estimation of EGCG. The optimized formula (F4) demonstrated more prolonged drug release and a significant improvement in the EE%, permeability, deformability and stability than the corresponding niosomes. The pharmacokinetic study investigated that F4 had a more sustained drug release and a higher bioavailability than the conventional niosomes and free drugs. Nanospanlastics could be a promising approach for improving the bioavailability of EGCG.

## 1. Introduction

Natural plants are an important and affordable source for valuable drugs that could be used effectively for the treatment of several diseases. Green tea is obtained from tea plant leaves (*Camilla sinensis*), which are widely used as a traditional health drink and is a vital source of various antioxidant polyphenols [1,2]. Green tea leaves have a number of valuable effects. However, black tea loses these effects as a result of the oxidation of its chemical components [3]. Catechins are the main polyphenolic constituents in green tea that are responsible for the majority of its anti-oxidant effects. Many studies have attributed the various valuable effects of green tea to (−) Epigallocatechin gallate (EGCG) [4,5]. EGCG is the most important green tea flavonoid and has attracted the interest of many researchers as a promising drug in the pharmaceutical, nutritional, and cosmetic fields. EGCG has exhibited many pharmacological activities such as anti-tumor, anti-oxidant, and anti-inflammatory effects [6,7,8,9]. Moreover, it has beneficial effects on cardiovascular diseases, diabetes, Parkinson’s disease, stroke, Alzheimer’s disease, and obesity [10,11]. However, it has limited bioavailability [12,13], which hinders its pharmacological applications and results in a significant inconsistency between the ex vivo and in vivo studies [13]. The poor bioavailability could be attributed to the difficulty in penetrating the cell membranes due to its high hydrophilicity [14]. Additionally, EGCG is unstable and susceptible to ambient conditions such as pH changes, oxygen, and other stress factors [13]. Using different formulation strategies for encapsulating EGCG is considered an efficient approach to combat these shortcomings [1,14,15].

The nanovesicular systems, such as liposomes and niosomes, are comprised of polar and non-polar parts; hence they could entrap both hydrophilic and lipophilic drugs. Nanovesicles can also improve both the stability and permeability of encapsulated drugs [2]. Additionally, they could deliver drugs to their target sites in a sustained or controlled manner that enhances their bioavailability [16]. Some studies demonstrate improving the stability and bioavailability of EGCG via encapsulation into nanovesicles. Zou et al. [17] reported that nanoliposomes could efficiently delay the decomposition of EGCG in vitro. Besides, Song et al. [14] explored that the niosomal formulations of (+) catechin and (−) EGCG exhibited higher stability, improved absorption, and lower toxicity than the free drug.

However, these conventional carriers have a non-flexible nature and lack deformability during passage through different biological membranes [18]. Hence, recent studies have discussed improving the deformability of these conventional nanovesicles in order to increase their permeability through different biological membranes [19]. Spanlastics are surfactant-based deformable nanocarriers that were formulated by Kakkar and Kaur [20]. The main components of spanlastics are non-ionic surfactants and EAs. EAs act as a destabilizing factor of the vesicular membranes of nanocarriers increasing their flexibility and permeability across the biological membranes [19,21] by squeezing through different pores of the biological layers without rupture. Nanospanlastics are non-immunogenic, biodegradable, and safe deformable nanovesicles. Moreover, they are more chemically stable than conventional liposomes [22]. Hence, nanospanlastics act as a stable nanocarrier that could encapsulate EGCG and increase its stability. Moreover, spanlastics can improve the poor permeability of EGCG due to their deformability by squeezing through narrow pores of the biological membranes [18,23]. The current study aimed to enhance the oral bioavailability of EGCG by encapsulation into spanlastic nanocarriers.

## 2. Results and Discussion

### 2.1. HPLC Method Development, Optimization and Validation

Method development and optimization were based on the achievement of suitable sensitivity, rapid analysis, appropriate peak parameters, and ease of operation in addition to the reasonable chromatographic separation of the studied components from the possible matrix contribution of formulation components [24].

**Figure 1:**
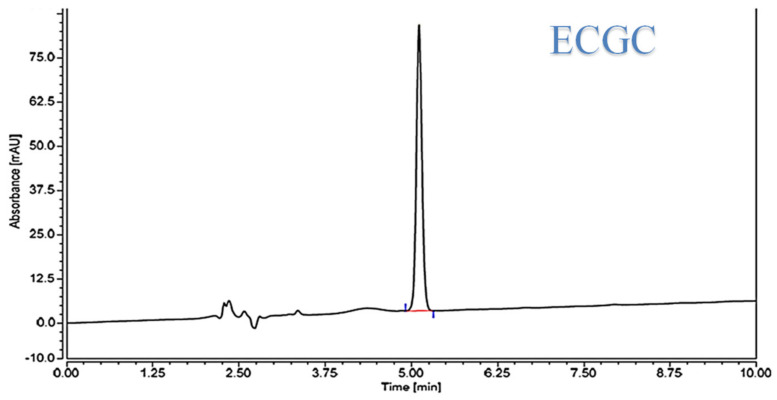
HPLC chromatogram of EGCG.

Firstly, methanol was selected as an organic modifier due to its availability and cheaper price with better peak shapes as well [25]. A mobile phase with low proportions of methanol was needed to enhance the separation parameters. A mobile phase with 75% water and 25% methanol achieved the best chromatographic characteristics for the ECGC peak with a reasonable retention time. The influence of pH of the mobile phase on the capacity factor (K′) of the ECGC peak was investigated in order to optimize HPLC parameters. The impact of pH was investigated using potassium dihydrogen phosphate buffer at a pH range of 3–6. pH 3 was chosen in order to improve the ECGC peak shape and to minimize its accompanied asymmetry. Eventually, a flow rate of 1 mL/min at an ambient temperature was selected to permit better elution time and reasonable peak separation from the combined matrix components with UV detection at 280 nm. The HPLC chromatogram exhibited a sharp peak with obvious baseline separations, Figure 1. The average retention time for ECGC was found to be 5.14 ± 0.12 min, for seven replicates.

The optimized method was validated according to the ICH guidelines [26]. The linearity of the current analytical method was granted by the high value of the correlation coefficient (0.999) and the small value of y-intercept (0.0093) and randomly distributed residuals, which demonstrates the linear relationship between the concentrations of EGCG and the peak areas.

The method precision was investigated by the low (<2%) coefficients of variation values (CV%) for repeatability and intermediate precision studies.

The LOD and LOQ provide an acceptable indication of the sensitivity of the validated technique [27].The detection and quantitation limits were calculated on the basis of the SD of the response approach and they were 35.80 and 108.42 ng/mL, respectively. The system suitability parameters were checked automatically using the Chromeleon 7 software and were found to be within acceptable ranges.

The method specificity was validated by the determination of peak purity factor, with the help of the diode array UV detector (DAD), and was found to be 999. The accuracy of the analytical technique is determined on the basis of the closeness of the obtained values to the true values. The accuracy of the present technique was performed by using known concentrations of ECGC (300, 600, and 900 ng/mL) and the results were compared with the expected results. The recovery study was conducted in triplicate (for each sample), and the percentage recovery was estimated. The excellent recoveries (95.96–98.12% with % RSD of 0.68–1.44%) suggested good accuracy of the proposed methods.

Moreover, the robustness of the HPLC method to minor changes in the optimized experimental conditions, such as the flow rate, was studied. The results demonstrated that the method was insensitive to the changes in flow rate. The stability of ECGC was also investigated in the mobile phase. The studied EGCG solutions exhibited no chromatographic changes for 3 days when stored refrigerated at 4 °C and for 24 h when kept at room temperature.

The above findings explored that the current optimized and validated HPLC method is a rapid, simple, precise, accurate, and robust technique that can be used for the quantification of EGCG.

### 2.2. Preliminary Screening Studies

The preliminary screening study was used for selecting the most suitable conditions and the most appropriate levels of different independent variables for preparing the spanlastic formulations. The effect of both the rotation speed and the time of sonication on EE% of EGCG-loaded SNVs was studied (Table S1). The results explored that the rotation speed had a significant negative impact on EE% (*p* < 0.01) that could be attributed to decreasing the size of EGCG-loaded SNVs by increasing the speed of rotation [18,28].

Additionally, sonication time had a significant negative influence (*p* < 0.001) on EE% of EGCG within the nanospanlastic vesicles. Increasing the sonication time to 5 min resulted in a significant decrease in EE% that could be interpreted on the basis of decreasing the size of the SNVs or escaping of EGCG to the external aqueous medium rather than entrapment within the SNVs during disruption and re-aggregation of the nanovesicles by sonication [29,30]. Regarding the ratio of non-ionic surfactant to EA, it was observed that the 1:1 ratio had the lowest EE% ((*p* < 0.01), Table S2). These findings could be explained on the basis of increasing the fluidization of the vesicular membrane, which results in increasing drug leakage from the nanospanlastic vesicles at lower concentrations of the non-ionic surfactant [31].

With respect to the type of non-ionic surfactant, Cremophor RH 40 exhibited the lowest EE% (*p* < 0.001). This may be attributed to its lower lipophilicity (HLB = 14–16) compared to Span 60 (HLB = 4.7) and Span 80 (HLB = 4.3), which in turn caused lower vesicle rigidity [32]. Concerning the type of EA, EGCG-loaded spanlastics containing Brij 35 had the lowest EE% (*p* < 0.01) compared to those containing Tween 60 and Tween 80. This might be attributable to its higher HLB value (16.9) and the shorter carbon chain length(C12) of Brij 35 [33].

According to the preliminary screening study, 3 min sonication time and 500 rpm rotation speed were selected for the fabrication of EGCG-loaded SNVs using the non-ionic surfactants (Span 60 and Span 80) and the EAs (Tween 80 and Tween 60) at 3:2 and 4:1 ratios of non-ionic surfactants to EA.

### 2.3. Analysis of the 2^3^ Factorial Design of EGCG-Loaded SNVs

Table 1 illustrates the composition and the responses (EE%, Q_12h_, and PS) of EGCG-loaded nanospanlastic formulations prepared according to the 2^3^ factorial design. Regression equations demonstrated the influence of various independent variables on the studied responses by comparing the factor coefficients. The positive sign, in front of the factor coefficients, showed a positive influence on the studied responses, whereas the negative sign demonstrated a negative impact [19,34].

In the present model, adequate precision values for Y1, Y2, and Y3 were found to be more than 4 (14.698, 32.553, and 15.996, respectively), Table 2. Hence, the chosen model could be used successfully for navigating the design space [23]. The predicted *R*^2^ was measured to determine the response value predictability. The adjusted *R*^2^ is the modified version of *R*^2^ that designates the goodness of fit of the chosen model to the observed data. The values of adjusted and predicted *R*^2^ statistics should be within about 0.20 of each other in order to be in reasonable harmony [35]. Table 2 demonstrates that the values of *R*^2^, predicted *R*^2^ and adjusted *R*^2^ for different responses are relatively high. Moreover, it is clear that the predicted *R*^2^ values of different responses were close to the adjusted *R*^2^ values. These outcomes showed that the obtained data are highly statistically valid and form an excellent fit to the obtained data.

Regression equations of different responses in terms of coded factors

(1) $$\mathrm{EE}\;\left(Y1\right)\;=+86.28+3.40\times X1-5.93\times X2+3.93\times X3$$

(2) $$Q12h\left(Y2\right)\;=+79.91+2.49\times X1+10.60\times X2-3.04\times X3$$

(3) $$\mathrm{PS}\;\left(Y3\right)\;=+324.63+60.83\times \;X1+20.57\times X2-36.04\times X3$$

**Figure 2:**
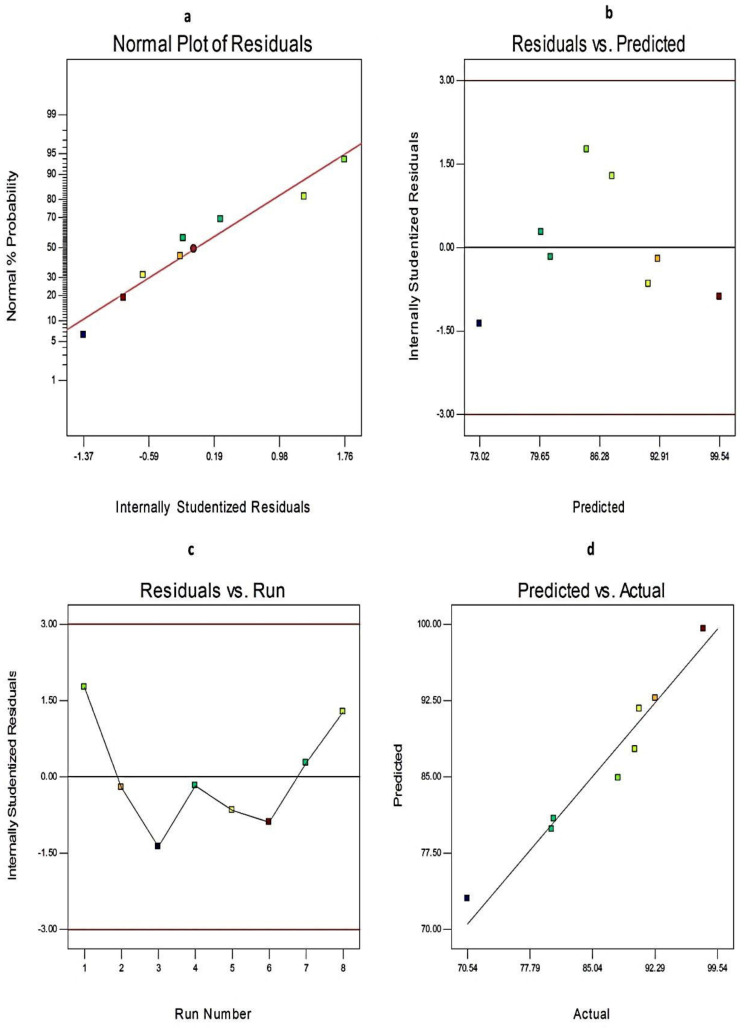
The diagnostic plots for EE% of EGCG-loaded spanlastics (**a**) plot of normal % probability versus the internally studentized residuals, (**b**) plot of the internally studentized residuals versus the predicted values, (**c**) plot of the internally studentized residuals versus the run number, and (**d**) plot of the predicted versus the actual values of EE%. Abbreviation: EE, entrapment efficiency of EGCG-loaded spanlastics.

**Figure 3:**
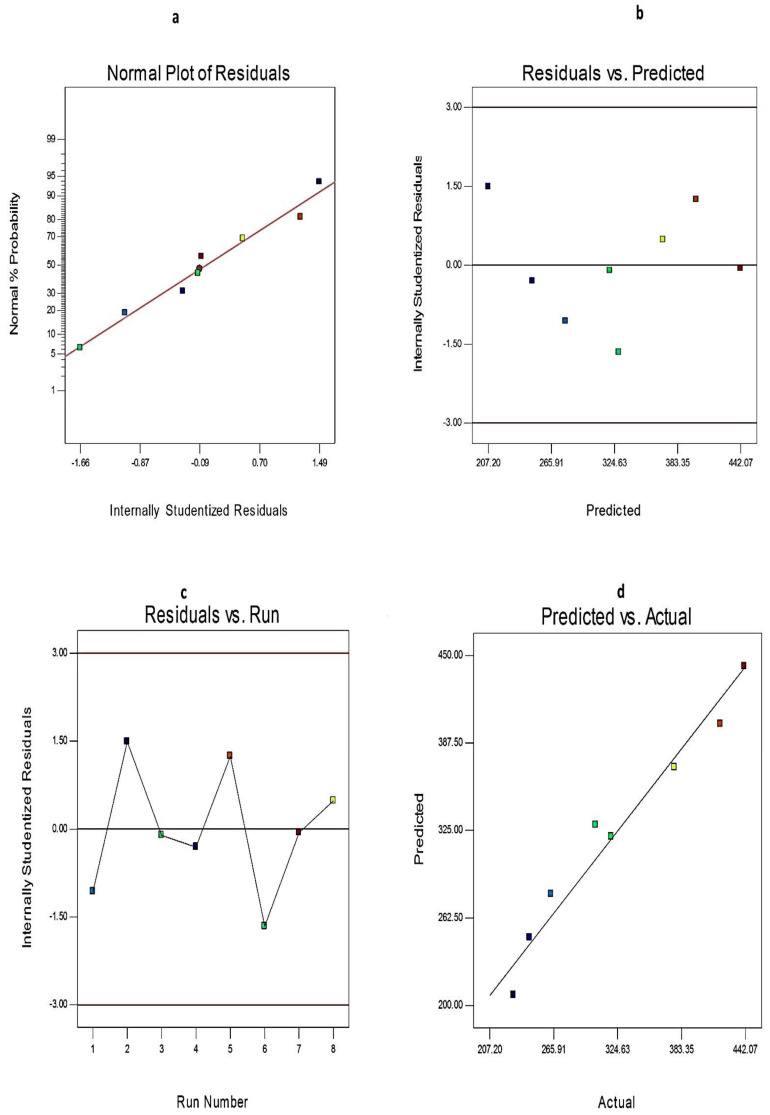
The diagnostic plots for PS of EGCG-loaded spanlastics (**a**) plot of normal % probability versus the internally studentized residuals, (**b**) plot of the internally studentized residuals versus the predicted values, (**c**) plot of the internally studentized residuals versus the run number, and (**d**) plot of the predicted versus the actual values. Abbreviation: PS, particle size of EGCG-loaded spanlastics.

**Figure 4:**
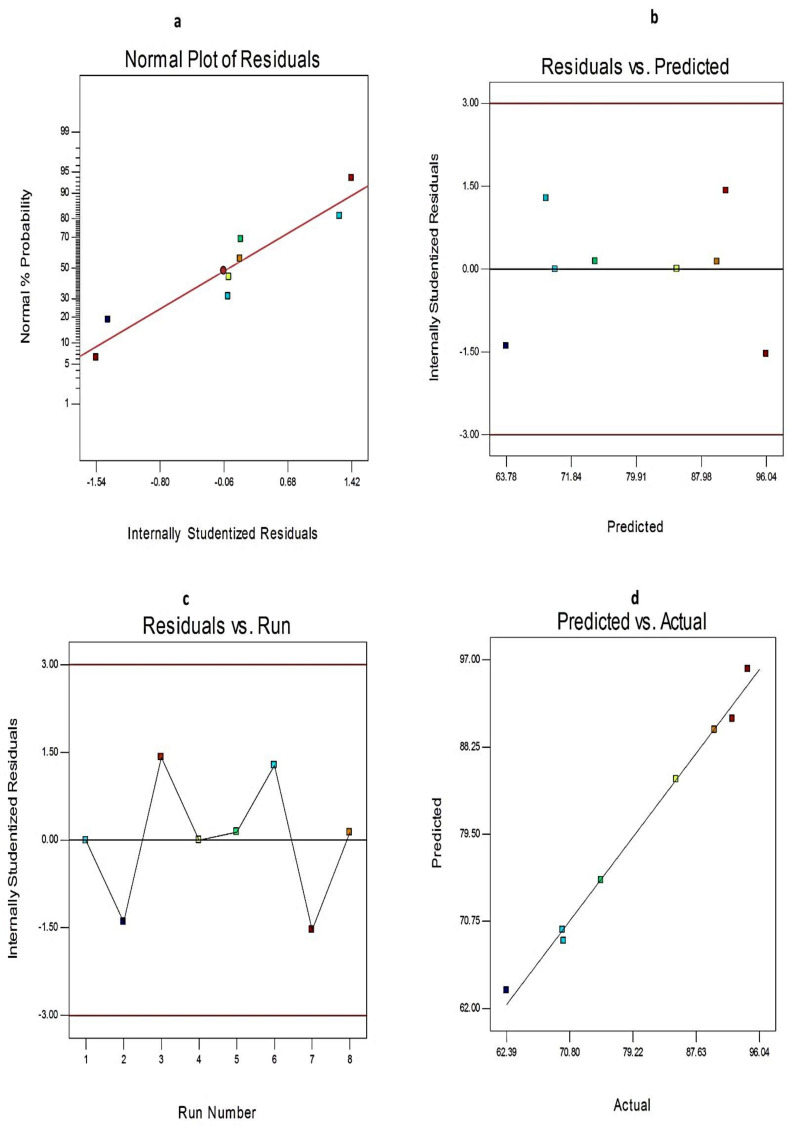
The diagnostic plots for Q_12h_ of EGCG-loaded spanlastics (**a**) plot of normal % probability versus the internally studentized residuals, (**b**) plot of the internally studentized residuals versus the predicted values, (**c**) plot of the internally studentized residuals versus the run number, and (**d**) plot of the predicted versus the actual values of Q_12h._ Abbreviation: Q_12h_, % EGCG released after 12 h.

Additionally, the credibility and the goodness of fit of the current model were investigated from the diagnostic plots for EE%, Q_12h,_ and PS of EGCG-loaded SNVs, Figure 2 and Figure 3, respectively. The difference between the actual and predicted values of the three responses was denoted as the residuals. Figure 2a, Figure 3a, and Figure 4a explore the normal probability plots of residuals as a linear pattern with a normal distribution of the residuals. Therefore, the data require no transformation. Figure 2b, Figure 3b, and Figure 4b demonstrate a plot of the residuals versus the predicted responses and show the absence of constant error because the color points, representing the values of different responses, were located within the limits near to the zero-axis and scattered randomly. Figure 2c, Figure 3c, and Figure 4c describe the residual versus run plots and exhibit a random and uniform scattering of points. Hence, no lurking variables affect the tested responses. Figure 2d, Figure 3d, and Figure 4d investigate the excellent analogy between the predicted and the observed values of Y1, Y2, and Y3 of EGCG-loaded SNVs in the present model.

ANOVA analysis (Table 3) was used to estimate the significance of different independent variables. *p*-value that is lower than the threshold level (0.05) demonstrates the significance of the model terms with rejection of the null hypothesis (H_0_) in favor of the alternative hypothesis.

#### 2.3.1. The Effect of Formulation Variables on EE% of EGCG-Loaded SNVs

The drug content (entrapped + un-entrapped) of EGCG-loaded SNVs ranged from 95.78 ± 1.22% to 98.76 ± 1.18%. EE% is a vital tool for estimating drug retention within the nanovesicles [36]. EGCG-loaded SNVs exhibited reasonable EE% that ranged from 70.53 ± 1.81% to 97.93 ± 1.28%, Table 1.

**Figure 5:**
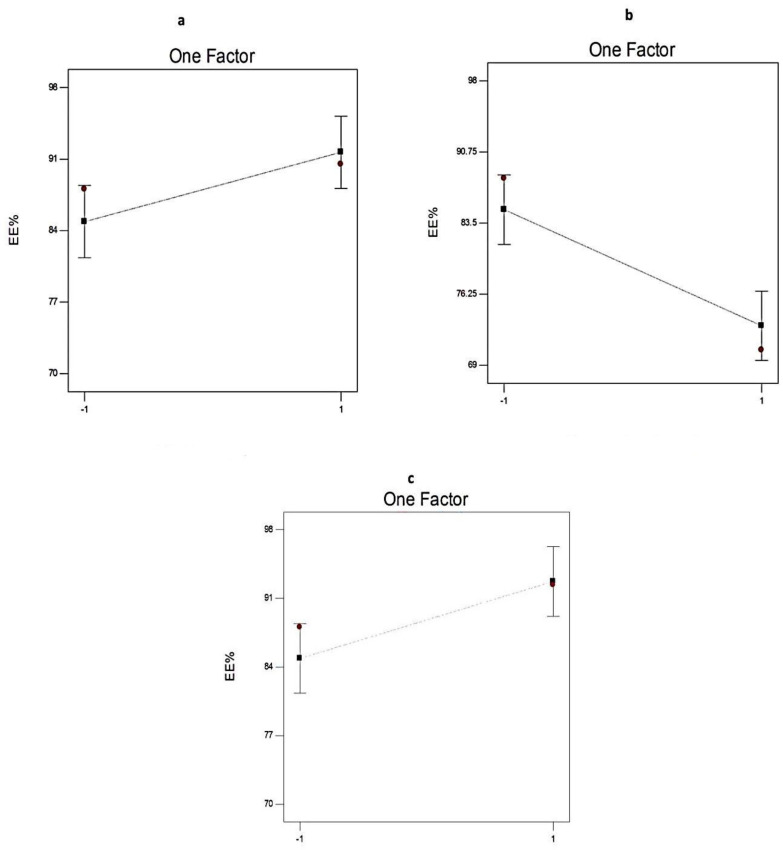
The effect of different independent variables (**a**) ratio of Span to EA, (**b**) type of non-ionic surfactant, and (**c**) type of EA on EE% of EGCG-loaded spanlastics according to 2^3^ factorial design. Abbreviation: EE, entrapment efficiency; EA, edge activator; EGCG, epigallocatechin gallate.

Figure 5 and Table 3 show the significant effect of the chosen independent variables (the ratio of Span: EA, type of the non-ionic surfactant, and type of EA) on the EE% of the EGCG-loaded SNVs.

Regarding the influence of the ratio of Span: EA (X1) on EE%, it is worth noting that X1 has a significant positive impact (*p* < 0.05) on drug entrapment within the SNVs. That could be attributed to increasing the rigidity of the vesicular membrane and accordingly, minimizing the leakage of the EGCG and improving the EE% [19].

With respect to the type of non-ionic surfactant (X2), Span 60-based spanlastic formulations have significantly higher EE% (*p* < 0.01) than Span 80-based SNVs. That could be explained on the basis of the saturated alkyl chain of Span 60 and its higher phase transition temperature (Tc = 53 °C) that result in decreasing the leakage of Span 60-based SNVs. However, Span 80 has a lower phase transition temperature of −12 °C [37] and a double bond in its alkyl chain that hamper the development of a tight vesicular membrane [38,39].

In addition, the type of EA (X3) had a significant impact (*p* < 0.05) on EE% of EGCG within the developed nanospanlastic vesicles. The EE% of SNVs containing Tween 60 was significantly higher than that of Tween 80-based nanospanlastic vesicles. That may be attributed to the unsaturation of the Tween 80 alkyl chain that results in increasing both the fluidity of the vesicular membrane and drug leakage [39].

#### 2.3.2. The Effect of Formulation Variables on Q_12h_ of EGCG-Loaded Spanlastics

The sink condition is a critical issue for conducting the in vitro release study [23]. The solubility of EGCG in phosphate-buffered saline (pH = 7.4) was found to be 5.07 ± 0.21 mg/mL. Therefore, the chosen receptor medium could achieve the required sink conditions because the sink condition is attained when the equilibrium solubility of drugs in the dissolution medium is at least 3 times the volume needed to attain the drug saturation.

**Figure 6:**
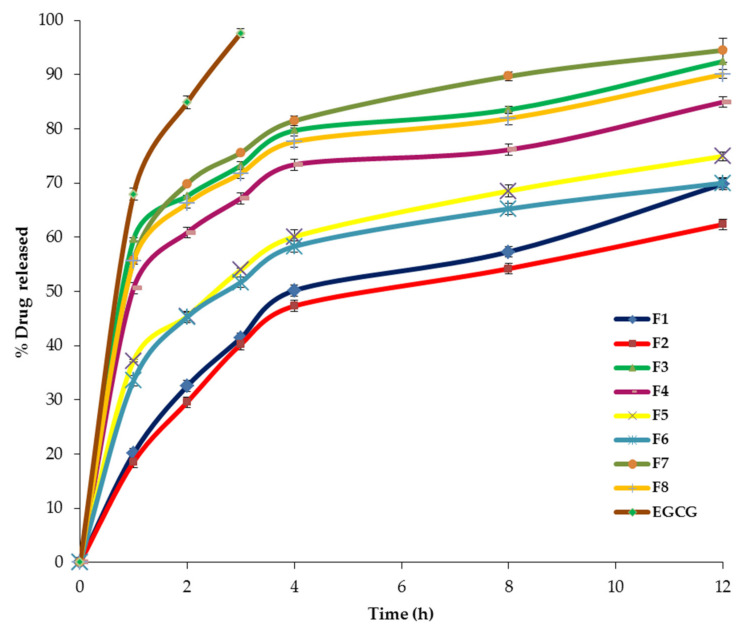
The in vitro release profile of EGCG-loaded spanlastics and EGCG dispersion for 12 h. Abbreviation: EGCG, epigallocatechin gallate.

The eight EGCG-loaded spanlastic formulations demonstrated a sustained release profile that varied from 62.39 ± 2.14% to 94.52 ± 1.07% after 12 h (Figure 6, Table 1). These findings explored that the nanospanlastic vesicles act as perfect reservoirs for EGCG [40]. However, the free EGCG exhibited a higher % drug released of 97.68 ± 1.33% after 3 h. That might be attributable to attaining successfully the sink conditions and the absence of hampering the in vitro release of EGCG by the cellulose membrane [41]. These outcomes agreed with Badria et al. [23], who demonstrated that sodium valproate-loaded spanlastics showed a more prolonged drug release than the free drug. Besides, Li et al. [27] reported that the in vitro release of EGCG-loaded niosomal formulations was more sustained than the free drug. Additionally, Safwat et al. [42] demonstrated that free EGCG diffused rapidly throughout the dialysis membrane. By contrast, EGCG-loaded gold nanoparticles exhibited sustained drug release for a prolonged period of time.

**Figure 7:**
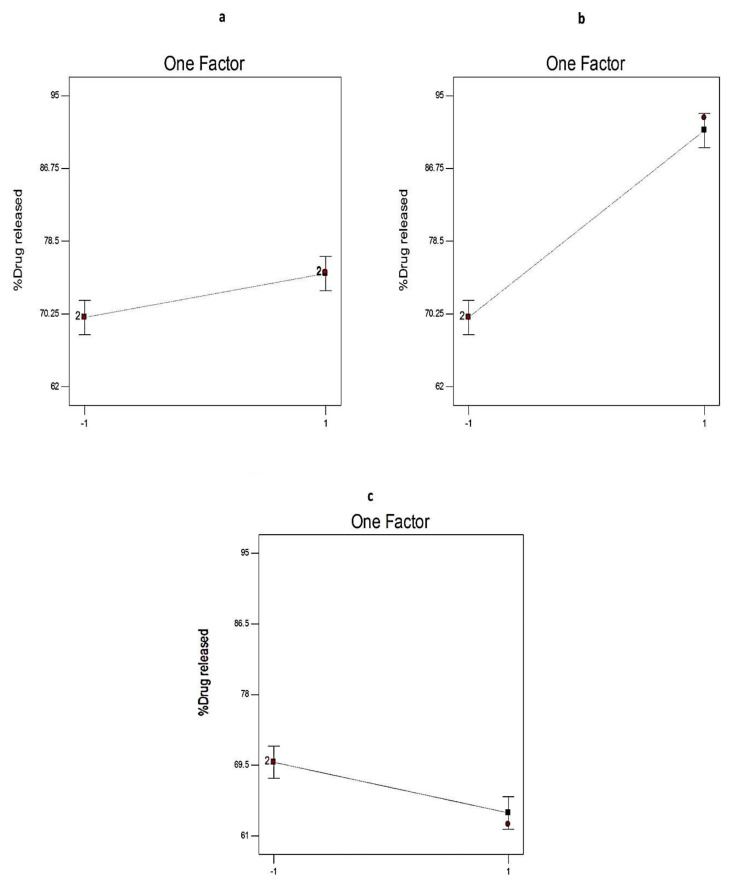
The effect of different independent variables (**a**) ratio of Span to EA, (**b**) type of non-ionic surfactant, and (**c**) type of EA on Q_12h_ of EGCG-loaded spanlastics according to 2^3^ factorial design. Abbreviation: Q_12h_, % drug released after 12 h; EA, edge activator; EGCG, epigallocatechin gallate.

Figure 7 and Table 3 illustrate the significant impact of different independent variables (X1, X2, and X3) on Q_12h_ of EGCG from SNVs.

The ratio of Span: EA (X1) showed a significant positive impact on Q_12h_ of the prepared nanospanlastics (*p* < 0.01) that may be due to the formation of mixed micelles, at high levels of EAs, that have a lower sensitivity to the concentration gradient than the vesicles [43].

In addition, it was noted that the Q_12h_ of Span 80-based SNVs was significantly higher than that in case of Span 60 (*p* < 0.0001). That could be attributable to the lower phase transition temperature of Span 80 (Tc = −12 °C) than Span 60 (Tc = 53 °C). Hence, Span 60-based vesicles have fewer permeable rigid bilayers than Span 80. This is also in agreement with the fact that at room temperature, Span 60 molecules are present in the ordered gel state in bilayer structures. However, Span 80 molecules are in the disordered liquid-crystalline state [44,45].

With respect to the type of EA, Q_12h_ of Tween 80-based SNVs was significantly higher than that in case of Tween 60 (*p* < 0.01). That may be explained on the basis of the unsaturation of the alkyl chain of Tween 80 that increases the chain fluidity and permeability [39].

According to the kinetic study (Table 4), the in vitro release profile of EGCG-loaded spanlastics fitted well with the Baker–Lonsdale model and that of EGCG dispersion followed by the Higuchi diffusion model, demonstrating a diffusion-based in vitro release mechanism [46]. The Baker–Lonsdale model [47] is established from the Higuchi model [48] to designate the drug release as a diffusion process that is based on the Fickian law.

#### 2.3.3. The Effect of Formulation Variables on PS of EGCG-Loaded SNVs

Table 1 shows that the PS of EGCG-loaded SNVs ranged between 229.0 ± 1.54 nm and 441.1 ± 2.03 nm. ANOVA results (Table 3) displayed that the ratio of Span: EA (X1), the type of non-ionic surfactant (X2), and the type of EA (X3) significantly affected the PS of the prepared EGCG-loaded SNVs.

**Figure 8:**
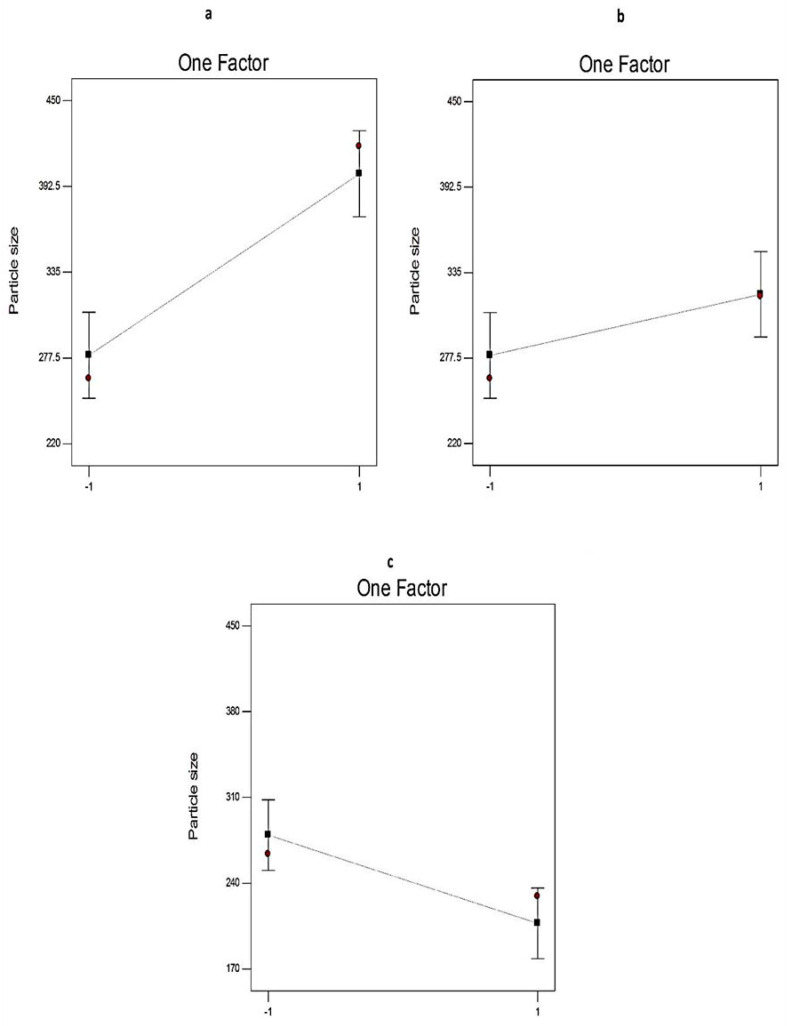
The effect of different independent variables (**a**) on the ratio of Span to EA, (**b**) type of non-ionic surfactant, and (**c**) type of EA on PS of EGCG-loaded spanlastics according to 2^3^ factorial design. Abbreviation: PS, particle size; EA, edge activator; EGCG, epigallocatechin gallate.

The influence of different independent variables (X1, X2, and X3) on the PS of the EGCG-loaded SNVs was investigated in Figure 8. Regarding the ratio of Span: EA (X1), the PS of the formulated EGCG-loaded nanovesicles decreased significantly (*p* < 0.01) upon increasing the concentration of EA. That may be attributable to facilitating the partition of particles as a result of reducing the interfacial tension at higher concentrations of EAs [49].

It is clear that the PS of the formulated EGCG-loaded SNVs was significantly (*p* < 0.05) affected by the type of non-ionic surfactant (X2). Span 80-based SNVs are of larger PS than Span 60 nanovesicles. This might be attributed to the development of monolayers of unsaturated Span 80 that are more expanded forming larger molecular areas [50].

The type of EA (X3) has a significant impact on the PS of the prepared EGCG-loaded SNVs. The PS of Tween 80-based SNVs was significantly (*p* < 0.01) larger than that of Tween 60-based vesicles. This could be attributed to the higher hydrophilicity of Tween 80 [51]. Using surfactants of a lower HLB (lower hydrophilicity) resulted in decreasing the surface energy and the development of nanovesicles with a smaller size [52]. These findings are in accordance with Ruckmani and Sankar who reported that Tween 80-based niosomes were larger in size than Tween 60-based vesicles [39] because the nanovesicle size increases as the hydrophilicity of the non-ionic surfactant increases.

The size distribution of the EGCG-loaded nanospanlastics was described in terms of PDI. As the PDI values approach zero, the homogeneity of the colloidal dispersion increases [19]. The prepared nanospanlastic dispersion demonstrated PDI values that ranged from 0.115 to 0.418, exploring good homogeneity of the EGCG-loaded nanospanlastic dispersions.

#### 2.3.4. The Optimization of EGCG-Loaded SNVs

The optimization process is used for the development of systematic strategies to get the best possible combinations required for preparing a high-quality pharmaceutical formula. It involves investigating the effect of different independent variables on the characteristics of pharmaceutical preparations with subsequent selection of the optimized formula [53].

The selection of the optimized EGCG-loaded nanospanlastic formula could be accomplished by means of simultaneous optimization of different variables [54] in the light of the desirability criteria [53]. Each response is described as a desirability value and the overall desirability value is estimated as the average of individual desirability values. The desirability value increases as it becomes closer to the target value [23,54]. The eight EGCG-loaded nanospanlastic formulations were optimized for the EE% (Y1), Q_12h_ (Y2), and PS (Y3). The optimization process aimed to maximize both EE% and Q_12h_ and minimize PS. The results demonstrated that F4 had the highest desirability value. Besides, the predicted values of F4 were 80.88%, 84.98%, and 248.33 for Y1, Y2, and Y3, respectively. The fitness of the current model, in choosing the optimized formula, was also investigated by the small percentage relative error (0.39, −0.002, and 1.82) for EE%, Q_12h_, and PS, respectively of F4 [31]. As a result, F4 could be considered as the optimized EGCG-loaded nanospanlastic formula.

### 2.4. Comparative Study of the Optimized EGCG-Loaded SNVs and the Conventional Niosomes

#### 2.4.1. Determination of EE%

The %EE of the optimized EGCG-loaded spanlastic formula (80.56 ± 1.57%) was significantly (*p* < 0.01) higher than the corresponding niosomal formula that has EE% of 69.84 ± 1.35%. These results are in accordance to Kakkar et al. [20] who found that the Ketoconazole-loaded spanlastic formula had a significantly higher EE% (68.82%) than the conventional niosomes (35.05%).

#### 2.4.2. In Vitro Release Study

**Figure 9:**
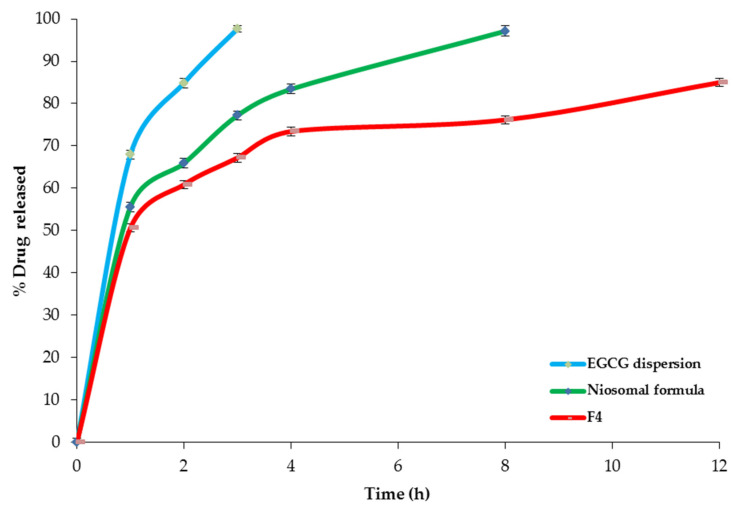
The in vitro release profile of F4, the conventional niosomes, and EGCG dispersion. Abbreviation: EGCG, epigallocatechin gallate.

Figure 9 demonstrates the in vitro release profiles of EGCG from the optimized spanlastic formula (F4) and the corresponding niosomal dispersion. It is clear that the in vitro release from optimized EGCG-loaded SNVs (84.98 ± 1.33% after 12 h) was more sustained than that from the conventional niosomal formula that exhibited 97.11 ± 1.24% drugs released after 8 h. That could be interpreted on the basis of better entrapment of EGCG within the SNVs. These findings are in agreement with ElMeshad et al. [52] who found that different Itraconazole-loaded spanlastic formulations exhibited more sustained drug release than the conventional niosomes. Both the spanlastic and the niosomal formulations demonstrated more sustained drug release than free EGCG dispersion that exhibited 97.68 ± 1.33% drugs released after 3 h.

#### 2.4.3. Ex Vivo Intestinal Permeation Study

**Figure 10:**
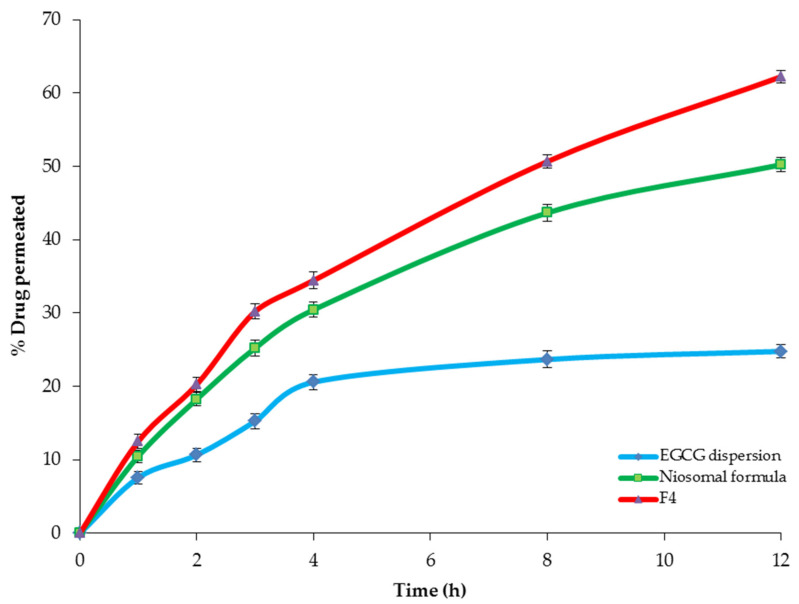
The ex vivo permeation profile of F4, the conventional niosomes, and EGCG dispersion for 12 h. Abbreviation: EGCG, epigallocatechin gallate..

The ex vivo intestinal permeation study investigated the effect of encapsulation of EGCG within the spanlastic and the niosomal nanovesicles on the permeation through the excised rat intestine. Table 5 and Figure 10 explore the permeation of EGCG from the optimized spanlastic formula (F4) and the niosomal formula in comparison with free EGCG dispersion. It is obvious that the optimized spanlastic formula (F4) showed significantly higher % drug permeated (62.22 ± 1.23%) than both the niosomal (50.22 ± 1.17%) and EGCG dispersion (24.77 ± 0.92 %) (*p* < 0.05 and *p* < 0.01, respectively) after 12 h. Moreover, Table 5 shows a significant enhancement of EGCG flux from both the optimized spanlastic formula and the niosomal formula than EGCG dispersion, with enhancement ratios of 6.76 and 4.73, respectively.

The higher permeation of EGCG-loaded SNVs could be explained on the basis of the higher deformability of spanlastic vesicles due to the presence of the EAs that increase the permeability of the SNVs across different biological membranes by squeezing through their narrow pores without rupture [55]. Additionally, the SNVs act as permeation enhancers that increase the drug permeability through biological membranes [35].

The kinetic analysis investigated that the permeation profiles of F4 and the niosomal formula followed the Baker–Lonsdale model, whereas EGCG dispersion followed the Higuchi model, Table 6.

#### 2.4.4. Measurement of Vesicle Deformability

The elasticity of the spanlastic vesicles describes their ability to permeate and squeeze through the narrow pores of biological membranes without rupture. This property distinguishes them from conventional niosomes [23]. The DI of the optimized EGCG-loaded SNVs (19.27 ± 1.2) was significantly (*p* < 0.0001) higher than that (1.48 ± 0.02) of the corresponding niosomes. This could be explained on the basis of increasing the flexibility of the lipid bilayer [19] by the addition of EAs.

The vesicle size of the spanlastic and the niosomal formulations was estimated before and after extrusion (Table 7). It is clear that EGCG-loaded SNVs retained their size after extrusion with no significant difference (*p* > 0.05) in the vesicle size. This could be attributed to increasing the deformability of the spanlastic vesicles after the addition of EA [56,57]. However, the significant decrease (*p* < 0.0001) in the vesicle size of EGCG-loaded niosomes, after extrusion, may be explained on the basis of the lack of deformability of the niosomal vesicles which results in rupturing the nanovesicles during passage through the nylon membrane [58].

#### 2.4.5. The Stability Study

According to the results of the stability test, it is obvious that the stored EGCG-loaded spanlastic formula (F4) demonstrated no significant difference (*p* > 0.05) in the EE%, drug content, Q_12h_, % drug permeated, and DI upon comparing with the fresh spanlastic formula (Table 8). Whereas, the stored niosomal formula exhibited a significant decrease in the drug content (*p* < 0.01), EE% (*p* < 0.01), Q_12h_ (*p* < 0.001), % drug permeated (*p* < 0.01), and DI (*p* < 0.001) when compared to the fresh formula. These results showed that nanospanlastics are more stable carriers than conventional niosomes. This could be explained on the basis of better drug encapsulation within the SNVs.

### 2.5. Characterization of the Optimized EGCG-Loaded Spanlastic Formula

#### 2.5.1. Morphological Characterization by SEM

**Figure 11:**
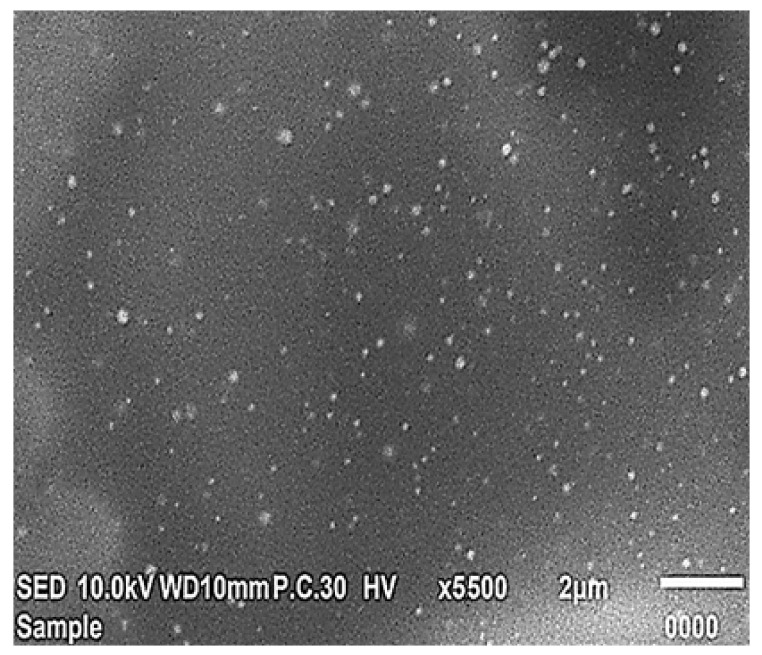
Scanning electron micrograph of the optimized EGCG-loaded nanospanlastic formula (F4). Abbreviation: EGCG, epigallocatechin gallate.

The optimized EGCG-loaded nanospanlastic formula (F4) appeared as a homogenous dispersion of well-identified spherical vesicles (Figure 11). It is possible to interpret the spherical shape of EGCG-loaded SNVs on the basis of the amphiphilicity of the non-ionic surfactants that results in their tendency to decrease the surface-free energy and spontaneous development of spherical nanospanlastic vesicles within the aqueous environment [59,60].

#### 2.5.2. Determination of Particle Size and Zeta Potential

**Figure 12:**
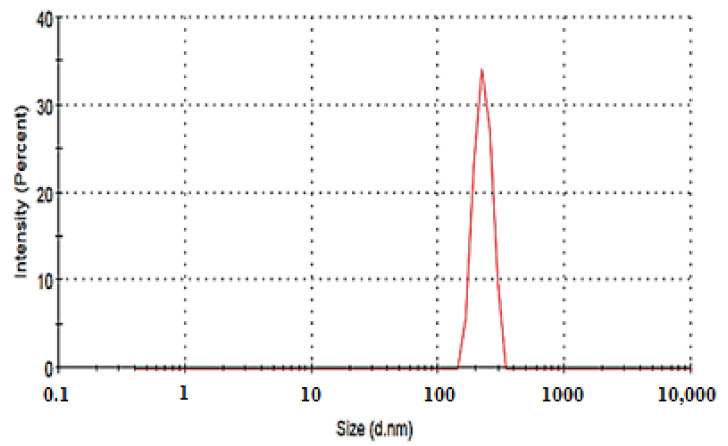
Particle size distribution curve of the optimized EGCG-loaded spanlastic formula (F4). Abbreviation: EGCG, epigallocatechin gallate.

The particle size of the optimized EGCG-loaded SNVs (F4) was found to be 243.8 ± 1.24 nm, with a PDI value of 0.21. The small value of PDI reveals the homogenous distribution of the particle size of EGCG-loaded SNVs, Figure 12.

High positive or negative zeta potential denotes the development of a stable colloidal dispersion because of the repulsion between the nanovesicles that permits the formation of a stable and non-agglomerated suspension [61]. The optimized EGCG-loaded spanlastic formula (F4) has a high negative zeta potential value (−41.23 mv) indicating the stability of the selected spanlastic formula. The reason for the negative zeta potential value of nanovesicles, containing non-ionic surfactants, was discussed by some researchers on the basis of the adsorption of counterions at the vesicular surface [62], the orientation of non-ionic surfactant hydroxyl groups with respect to the aqueous medium [63], and the ionization of free groups present on the surface of nanovesicles [64].

The high cellular uptake of the negatively charged nanocarriers, without any repulsion with the negatively-charged cell membrane, might be due to the non-specific adsorption and development of clusters of nanovesicles on the cellular membrane [65].

### 2.6. Pharmacokinetic Study

**Figure 13:**
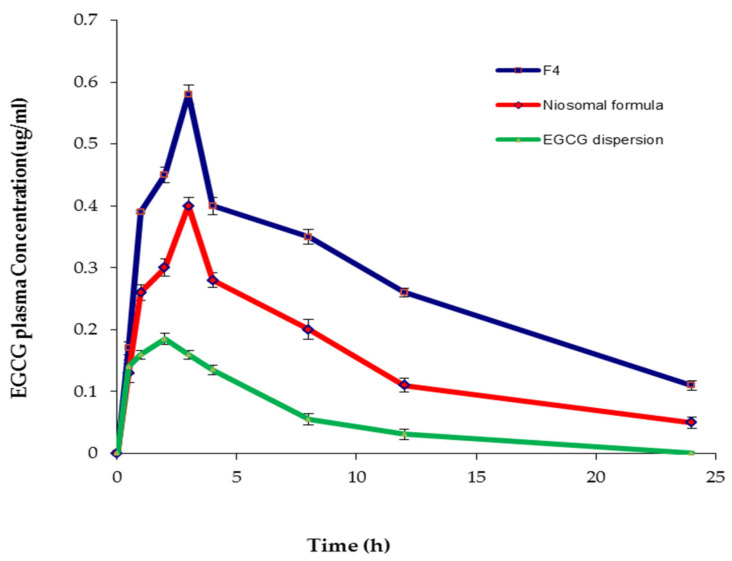
The plasma concentration-time curve of F4, the conventional niosomes, and EGCG dispersion. Abbreviation: EGCG, epigallocatechin gallate.

The pharmacokinetic study was conducted in order to monitor the effect of encapsulating EGCG into the spanlastic and niosomal carriers on its bioavailability. The absorption of EGCG was tracked in rats by plotting plasma concentrations against time, as shown in Figure 13. Table 9 demonstrates different pharmacokinetic parameters. The order of increasing the t_1/2_ was arranged as follows: EGCG-loaded SNVs > niosomal formula > EGCG dispersion. These findings exhibited the sustained release of EGCG from the spanlastic vesicles. Consequently, EGCG-loaded SNVs could maintain a sustained drug concentration in the plasma, which resulted in a reduction in the frequency of administration and improving patient compliance. Cai et al. [13] reported that the controlled release of EGCG is a feasible method to increase its bioavailability because free EGCG has too short t_1/2_ to exert efficient clinical effects. Moreover, Safwat et al. [42] concluded that the ability of nanoparticles to hold EGCG for a sustained period of time is a crucial asset to increase the circulation time of the drug in the blood and to deliver it effectively to remote areas.

Additionally, the results showed that EGCG-loaded SNVs exhibited significantly higher C_max_ than the corresponding niosomal formula (*p* < 0.05) and the free EGCG dispersion (*p* < 0.001). The higher C_max_ of EGCG that attained post-administration of EGCG-loaded spanlastic formulation investigated the enhancement of EGCG absorption by loading into the SNVs.

The improvement in the oral bioavailability was also investigated from the results of the area under the curve (AUC_0–24_) that showed a significant increase in the case of the optimized EGCG-loaded spanlastic formula than the conventional niosomal formula (*p* < 0.01) and EGCG dispersion (*p* < 0.001). Besides, the % relative bioavailability (F_re_%) of EGCG-loaded spanlastics, in relation to free drug dispersion, was significantly (*p* < 0. 01) higher than that of the corresponding niosomes.

The pharmacokinetic test, thus, supported the ex vivo intestinal permeation study of increasing permeability and then bioavailability of EGCG-loaded SNVs. Improved absorption and higher plasma concentration of EGCG after administering spanlastic and niosomal formulations may be attributed to improving the permeation of EGCG due to the presence of the basic nanocarrier constituents (surfactants) that act as permeation enhancers [66]. Moreover, entrapping EGCG into nanocarriers increases its stability by protection from enzymatic degradation [13]. These outcomes are in close agreement with Dube et al. [67], who reported that the mechanism of improving the absorption of catechins is the enhanced stabilization of drugs after encapsulation within nanoparticles. Additionally, Hu et al. [68] reported that a self-double-emulsifying solid formulation could improve the oral bioavailability and C_max_ of EGCG due to the protection of EGCG from the enzymatic degradation and alkaline/neutral conditions via encapsulation of drug and sustained drug release.

Additionally, the higher bioavailability of EGCG-loaded spanlastics could be explained on the basis of higher deformability and elasticity of SNVs due to the presence of EAs that increase the fluidity of the vesicular membrane and permit their passage through narrow pores of the biological membranes [23]. Accordingly, nanospanlastics not only improve the stability of EGCG, but also increase the membrane permeability due to their deformability.

## 3. Materials and Methods

### 3.1. Materials

EGCG, PEG-40 hydrogenated castor oil (Cremophor RH 40), polyoxyethylene (23) lauryl ether (Brij 35), and methanol (HPLC grade) were purchased from Sigma Chemical Co. (St. Louis, MI, USA). Sorbitan monostearate (Span 60), Sorbitan monooleate (Span 80), and cholesterol (CHOL) were obtained from Oxford Lab Chemicals (Mumbai, India). Potassium dihydrogen phosphate and potassium monohydrogen phosphate were provided from Alpha Chemica (Mumbai, India). Polyoxyethylene (20) sorbitan monooleate (Tween 80), absolute ethyl alcohol, and polyoxyethylene (20) sorbitan monostearate (Tween 60) were obtained from El-Nasr Chemical Co. Company (Cairo, Egypt). Spectra/Pore^®^ dialysis membranes (Molecular weight cut-off 12,000–14,000 Dalton) were obtained from Spectrum Laboratories Inc. (Rancho Dominguez, CA, USA). All other chemicals, solvents, and reagents were of analytical grade.

### 3.2. Methods

#### 3.2.1. HPLC Analysis of EGCG Using a Newly Developed and Validated RP-HPLC Method of Instrumentation

Dionex UltiMate 3000RS HPLC system (Thermo ScientificTM, DionexTM, Sunnyvale, CA, USA). It comprised of an LPG-3400RS quaternary pump, a WPS-3000RS autosampler, an Inertsil reversed-phase C18 (150 × 4.6 mm × 5 μm) column, a TCC-3000RS column thermostat, and a DAD-3000RS diode array detector. Data collection and processing were performed by Chromeleon 7 software.

##### Chromatographic Parameters

A new simple and robust RP-HPLC method was developed and validated for EGCG quantification. The mobile phase used was 0.05 mM potassium dihydrogen phosphate buffer (pH 3) and methanol at a 75:25 volume ratio. The injection volume was 20 μL and the elution was performed at a flow rate of 1 mL/min. All assays were performed at room temperature. The detection wavelength of EGCG was 280 nm.

##### Preparation of Standard and Calibration Solutions of EGCG

EGCG (50 mg) was dissolved in 50 mL methanol with the aid of bath sonication (ultrasonic bath, Elmasonic E 30 H, Elma, Schmidbauer GmbH, Singen, Germany) for preparing the standard stock solution. The EGCG standard working solutions, in the concentration range of 100–1000 ng/mL, have been prepared by diluting the stock standard solution with the mobile phase [69]. For each drug concentration level, triplicate injections of 20 μL were chromatographed under the conditions described above. To avoid photodecomposition, the stock and working standard solutions of EGCG were kept away from light.

##### Assay Validation

The developed and optimized HPLC method was validated as guided in the ICH Q2B guidelines [70]. The following parameters of the current technique were validated: linearity, accuracy, precision, selectivity, the limit of detection (LOD), and the limit of quantification (LOQ), system suitability, and robustness [71]. In addition, the stability of ECGC in the selected mobile phase was also evaluated by keeping the standard solutions of EGCG away from the light, in tightly capped containers, on a laboratory bench, and in the refrigerator [72].

#### 3.2.2. Preliminary Screening Studies

The preliminary screening studies were divided into two sections. The first section was conducted in order to choose the proper conditions (rotation speed and the sonication time) for the fabrication of EGCG-loaded spanlastics. EGCG-loaded spanlastic nanovesicles (SNVs) have been prepared using Span 60 as the non-ionic surfactant and Tween 80 as the EA at a 3:2 ratio at different rotation speeds (500 and 1000 rpm) for different times of sonication (3 and 5 min), Table S1. The second section of the preliminary test aimed to choose the most suitable levels of different variables for the preparation of EGCG-loaded nanospanlastics. EGCG-loaded SNVs were formulated using three types of non-ionic surfactants (Span 60, Span 80, and Cremophor RH 40), three types of EAs (Tween 80, Tween 60, and Brij 35) at three ratios of non-ionic surfactant to EA (1:1, 3:2 and 4:1) (Table S2). The prepared spanlastic formulations were evaluated with respect to their EE% that denotes the integrity of the vesicular membrane and the absence of drug leakage.

#### 3.2.3. Preparation of EGCG-Loaded Spanlastics

Table 1 investigates the composition of different EGCG-loaded spanlastics. All excipients used for fabricating nanospanlastic formulations of EGCG are approved by the FDA [73] and generally recognized as safe (GRAS) [74]. A number of non-ionic SAAs were selected to be used as the membrane-forming lipids and EAs because they have considerable advantages over other classes of surfactants with respect to safety, compatibility, and stability [20]. EGCG-loaded spanlastics were fabricated by the ethanol injection method [75] due to its simple and reproducible technique [23,76]. EGCG and the non-ionic surfactants were dissolved carefully into 3 mL of absolute ethanol. The ethanolic solution was injected carefully into a preheated aqueous medium (60 °C) containing the EA and stirred continuously on a magnetic stirrer (Jenway 1000, Jenway, UK) till the formation of milky spanlastic dispersion (10 mL). For complete evaporation of any remaining alcohol, the spanlastic dispersion was stirred for another 1h. The EGCG-loaded spanlastic dispersions were then subjected to bath sonication (water-bath ultrasonicator, Elmasonic E 30 H, Elma, Schmidbauer GmbH, Singen, Germany). The spanlastic formulations were then stored at 4 °C overnight for complete maturation of SNVs to be used for further studies.

#### 3.2.4. Experimental Design

A 2^3^ factorial design was performed using Design-Expert software, v7.0.0 (Stat-Ease, Inc., Minneapolis, MN, USA) to optimize the EGCG-loaded nanospanlastics [77]. The independent variables were the ratio of non-ionic surfactant: EA (X1), type of non-ionic surfactant (X2) and type of EA (X3). Dependent variables were the entrapment efficiency (EE %, Y1), the percentage of drug released after 12 h (Q_12h_, Y2), and the particle size (PS, Y3). Each factor was screened at two levels; the lower level (−1) and the upper level (+1), respectively.

The coefficient of determination (R^2^) and predicted/adjusted R^2^ of the chosen model were calculated for exploring its goodness of fit to the experimental results. Additionally, the significance level of the studied terms was investigated by the analysis of variance (ANOVA) [78].

#### 3.2.5. In Vitro Characterization of EGCG-Loaded Nanospanlastics

##### Determination of Drug Content and Entrapment Efficiency of EGCG-Loaded Nanospanlastics

The EE% of EGCG-loaded SNVs was determined using ultracentrifugation according to the indirect method in order to separate the un-entrapped drug, and 1 mL of EGCG-loaded SNVs was centrifuged at 20,000 rpm at 4°C for 1 h (Cooling centrifuge, HermleLabortechnik GmbH, Germany). The supernatant was passed carefully through a 0.20 µm pore size nylon membrane filter (Nylon Acrodisc, Gelman Sciences Inc., Ann Arbor, MI, USA) and the concentration of free EGCG was measured by HPLC at 280 nm. EE% was calculated as follows [19]:

(4) $$\mathrm{EE}\left(\%\right)\;=\;\left(\mathrm{Total}\;\mathrm{amount}\;\mathrm{of}\;\mathrm{EGCG}\;-\mathrm{Amount}\;\mathrm{of}\;\mathrm{free}\;\mathrm{EGCG}\right)\;\times \;100/\mathrm{Total}\;\mathrm{amount}\;\mathrm{of}\;\mathrm{EGCG}$$

The total drug content of EGCG (unentrapped + entrapped) was estimated via disrupting the spanlastic dispersions (1 mL) using isopropyl alcohol (100 mL) [79]. The samples were filtered, diluted, and analyzed for drug content using HPLC at 280 nm.

##### In Vitro Release Study of EGCG-Loaded SNVs

Prior to conducting the in vitro release test, the solubility of EGCG was estimated in phosphate-buffered saline (pH = 7.4) using the shake-flask method [80] to ensure attaining sink conditions of the chosen dissolution medium.

The membrane diffusion technique was used for studying the in vitro release profile of EGCG-loaded nanospanlastics [79]. The semi-permeable cellulose membrane was prehydrated carefully using a phosphate-buffered saline solution (pH = 7.4) at room temperature for 24 h. The cellulose membrane was fixed, using a rubber band, at the end of a glass cylinder attached to the dissolution apparatus shaft (USP apparatus II, Erweka DT-720, Kreuzau, Germany). The receptor medium was 300 mL phosphate-buffered saline solution of pH = 7.4 [81]. The receptor medium was stirred at 50 rpm and kept at 37 ± 0.5 °C. 1 mL of the spanlastic formulations containing entrapped EGCG or the equivalent concentration of EGCG dispersion were introduced in the donor chamber over the cellulose membrane; 200 µL aliquots were withdrawn at the predetermined time intervals and substituted by an equal volume of fresh buffer solution to preserve a constant volume of the receptor medium [77]. The withdrawn samples were filtered through a 0.20 µm nylon membrane filter (Nylon Acrodisc, Gelman Sciences Inc., Ann Arbor, MI, USA), diluted and analyzed using HPLC at 280 nm. Triplicate experiments were performed. The results are expressed as mean values ± SD. The mechanism of the in vitro release of EGCG from the SNVs was determined by a kinetic study using different mathematical models [38].

##### Determination of the Particle Size (PS) and Polydispersity Index (PDI) of EGCG-Loaded SNVs

The colloidal characters of the EGCG-loaded nanospanlastic dispersions were investigated by determination of PS and PDI. The EGCG-loaded nanospanlastic formulations (1 mL) were diluted by deionized water (100 mL) in order to get a reasonable scattering intensity. Then, the vesicle size and PDI of EGCG-loaded SNVs were determined using a particle size analyzer (NICOMP 380 ZLS Zeta Potential/Particle Sizer, Santa Barbara, CA, USA) [23,31]. Different measurements were performed in triplicate and the mean of three runs were determined.

#### 3.2.6. Statistical Optimization of EGCG-Loaded SNVs

The desirability values were used to select the optimized formula because they describe the closeness of the chosen responses to their optimal values [82]. The formulation that has the highest desirability value is selected as the optimized formula [23]. In the present study, the optimized formula was selected on the basis of maximum EE%, maximum Q_12h_, and minimum PS. Additionally, the optimized EGCG-loaded spanlastic formula was validated by calculating % relative error via comparing the observed responses (Y1, Y2, and Y3) with their corresponding predicted values [23] as follows:

% Relative error = (predicted value-observed value) × 100/predicted value(5)

The optimized EGCG-loaded nanospanlastic formula was subjected to a comparative study with the corresponding niosomes and evaluated by additional characterization tests.

#### 3.2.7. Comparative Study of the Optimized EGCG-Loaded Nanospanlastic Formula and the Conventional Niosome

##### Preparation of EGCG-Loaded Conventional Niosomes

The conventional EGCG-loaded niosomes were fabricated by the ethanol injection technique [56,83] using the same previous steps described for preparing the spanlastic dispersion without the addition of EA and with the addition of CHOL to the non-ionic surfactant at a 1:2 molar ratio.

##### Evaluation of EE%, Drug Content and the In Vitro Release Study

The EE%, drug content, and the in vitro release of EGCG-loaded niosomes were studied as previously described in the in vitro characterization section of spanlastics.

##### Ex Vivo Intestinal Permeation Study

The ex vivo intestinal permeation test was performed using 200–220 g Male Wistar rats after approving the study protocol by the committee of ethics, the Faculty of Pharmacy, Kafrelsheikh University, Egypt (Approval number KFS-2020/02) and according to the ethical guidelines [84,85,86]. After sacrificing rats under anesthesia, the small intestines were carefully removed and rinsed thoroughly with saline solution (0.9%) for removal of any mucosal or luminal contents [87,88].

The ex vivo permeation profile of the optimized EGCG-loaded nanospanlastic formula was compared with the corresponding niosomal dispersion and the aqueous dispersion of EGCG. The receptor medium was 300 mL phosphate-buffered saline solution of pH = 7.4 [81] that stirred at 50 rpm and kept at 37 ± 0.5 °C. The excised intestinal tract was cut into sacs that were filled with the tested formulations (1 mL). The two sides of the intestinal sac were tied with a surgical thread and attached to the shafts of the dissolution apparatus [87,89]; 200 µL sample was withdrawn at the predetermined time intervals (1, 2, 3, 4, 8, and 12 h) and replaced by the same volume of fresh buffer. Samples were passed through a 0.20 µm pore size nylon membrane filter (Nylon Acrodisc, Gelman Sciences Inc., Ann Arbor, Michigan, USA), diluted and analyzed using HPLC at 280 nm. The experiment was conducted in triplicate and the % EGCG permeated was described as the average ± SD. The ex vivo permeation graphs were plotted by comparing % EGCG permeated with respect to time.

Different pharmacokinetic parameters were estimated such as the steady-state flux of EGCG (J_ss_), permeability coefficient (K_p_), and the enhancement ratio (ER) of both EGCG-loaded spanlastic and niosomal formulations [77]. The mechanism of the ex vivo intestinal permeation of EGCG was determined by a kinetic study using different mathematical models [38].

##### Measurement of Vesicle Deformability

The deformability of the optimized nanospanlastic formula and the conventional niosomes was demonstrated by extrusion through a 100 nm nylon membrane filter for 5 min at 2.5 bar [90]. The vesicle size (before and after extrusion) of both the spanlastic and niosomal formulations was determined by a NICOMP 380 ZLS Zeta Potential/Particle Sizer (PSS Nicomp, Santa Barbara, CA, USA). The deformability index (DI) was calculated using the following equation:

(6) $$\mathrm{DI}=J\;{\left(r_{v}/r_{p}\right)}^{2}$$

where J is the amount of the extruded EGCG-loaded spanlastic/niosomal dispersion, r_v_: the vesicle size of the extruded EGCG-loaded spanlastic/niosomal formula and r_p_: the pore size of the nylon membrane filter.

The deformability, in terms of DI, was estimated by the extrusion method in order to compare the elasticity of the optimized EGCG-loaded SNVs with that of the conventional niosomal dispersion. This test could demonstrate the effect of EAs on the elasticity of spanlastic vesicles that permit them to pass through narrow pores of biological membranes. Besides, the vesicle size of both formulations was estimated before and after extrusion to investigate the effect of adding EAs on retaining the vesicular size after extrusion that prevents the rupture of the SNVs during passage through different membranes.

##### The Stability Test

Both the optimized EGCG-loaded spanlastic formula and the corresponding niosomal dispersion were kept at 4–8 °C in tightly closed glass vials for three months [40]. The EGCG-loaded spanlastic and the niosomal formulations were evaluated with regard to their EE%, drug content, Q_12h_, % drug permeated, and DI to investigate the influence of storage conditions on the stability of both formulations.

#### 3.2.8. Characterization of the Optimized EGCG-Loaded SNVs

##### Scanning Electron Microscopy (SEM)

The optimized EGCG-loaded nanospanlastic formula (1 mL) was suitably diluted using deionized water (100 mL) and affixed carefully onto the SEM sample aluminum stub with a sticking carbon tape. Afterward, the EGCG spanlastic dispersion was subjected to vacuum drying overnight followed by coating with gold film. After coating, the EGCG-loaded spanlastic vesicles were examined by scanning electron microscope (JSM-6360, JEOL, Tokyo, Japan) [91].

##### Determination of Particle Size and Zeta Potential

The optimized EGCG-loaded nanospanlastic dispersion (1 mL) was suitably diluted using deionized water (100 mL). The particle size and zeta potential of SNVs have been estimated, on the basis of a light scattering technique, using a particle size analyzer (NICOMP 380 ZLS Zeta Potential/Particle Sizer, Santa Barbara, CA, USA) via detection of the electrophoretic mobility of EGCG-loaded SNVs within the electric field [31,92].

#### 3.2.9. Pharmacokinetic Study

Male Wistar rats (200–220 g) were used in the pharmacokinetic study to compare the in vivo performance of EGCG-loaded SNVs with that of the corresponding niosomes and the free EGCG dispersion. The rats were obtained from the National Research Center (Dokki, Giza, Egypt) and kept in sawdust-bedded cages within a pathogen-free facility. The animal rooms were maintained at 50% relative humidity, at a controlled temperature of 25 ± 2 °C, and under 12 h-light, 12 h-dark cycles. Adaptation of the tested rats was conducted, under standard conditions, within the animal house for 14 days before performing the experiment. The experimental protocol and animals used were approved by the Committee of Ethics, Faculty of Pharmacy, Kafrelsheikh University, Egypt (Approval number KFS-2020/08). The methodology of the pharmacokinetic study was in accordance with the ethical guidelines [84,85,86].

Rats were randomly divided into three groups (6 rats each). Before performing the experiment, the rats were fasted overnight with free access to water. Group A administered EGCG-loaded spanlastic dispersion, group B administered the corresponding niosomal dispersion, and group C administered EGCG dispersion. Each dispersion, equivalent to 50 mg/kg of EGCG, [93,94], was administered orally by a gavage tube. Blood samples were withdrawn, from the tail vein, into heparin-containing tubes, immediately prior to dosing (zero time) and then after administration of EGCG formulations at 0.5, 1, 2, 3, 4, 8, 12, and 24 h. Different blood samples were centrifuged at 15,000 rpm for 10 min and 100 μL plasma samples were then collected, vortex-mixed for 1 min, and centrifuged at 5000 rpm for 5 min. The drug concentration was estimated using an HPLC assay at 280 nm and different pharmacokinetic parameters were determined using the plasma concentration-time curve, such as the maximum plasma concentration (C_max_), the area under the curve (AUC_0-24_), and the half-life (t_1/2_). The % relative bioavailability (F_rel_) of the niosomal and spanlastic dispersions was calculated in relation to free EGCG dispersion [95].

#### 3.2.10. Statistical Analysis

SPSS-11 software (SPSS. Inc., Chicago. IL, USA) was used for the statistical analysis of the results. The data, obtained from the 2^3^ factorial design, were analyzed using ANOVA by Design-Expert software (Stat-Ease, Inc., Minneapolis, Minnesota, USA) to investigate the effect of the selected variables on EE%, Q_12h,_ and PS of EGCG-loaded SNVs. *p*-values lower than 0.05 denote statistically significant variations between the tested terms.

## 4. Conclusions

The current study demonstrated the preparation of EGCG-loaded spanlastics as an effective and flexible nanocarrier for improving the bioavailability of EGCG. Eight EGCG-loaded nanospanlastics were effectively fabricated using the ethanol injection method according to 2^3^ factorial design. The optimized spanlastic formulation (F4) was chosen according to the desirability criteria on the basis of maximizing both EE% and Q_12h_ and minimizing PS. Using the simple and validated RP-HPLC method helped in rapid and robust determination of ECGC. Upon comparing with the conventional niosomes, F4 demonstrated more prolonged drug release, with higher EE%, deformability, stability, and bioavailability. In brief, these outcomes exhibited that EGCG-loaded spanlastics are a promising and flexible nanocarrier that could overcome the shortcomings of the limited permeation and poor stability of EGCG.

## Figures and Tables

**Table 1:** The 2^3^ factorial design and the composition of EGCG-loaded spanlastics.

Formula	Variables					
	Independent			Dependent		
	X1	X2	X3	Y1 *	Y2 *	Y3 *
F1	−1	−1	−1	88.04 ± 2.82	69.85 ± 1.33	263.6 ± 1.62
F2	−1	−1	1	92.35 ± 1.34	62.39 ± 2.14	229.0 ± 1.54
F3	−1	1	−1	70.53 ± 1.81	92.47 ± 1.28	318.8 ± 1.48
F4 #	−1	1	1	80.56 ± 1.57	84.98 ± 1.33	243.8 ± 1.24
F5	1	−1	−1	90.49 ± 1.22	74.97 ± 1.36	419.2 ± 1.82
F6	1	−1	1	97.93 ± 1.28	70.02 ± 1.25	304.5 ± 1.55
F7	1	1	−1	80.32 ± 1.38	94.52 ± 1.07	441.1 ± 2.03
F8	1	1	1	89.99 ± 2.16	90.08 ± 1.46	377.0 ± 1.93
Independent variables				Low (−1)	High (+1)	
X1: Ratio of Span to EA				3:2	4:1	
X2: Type of non-ionic surfactant				Span 60	Span 80	
X3: Type of EA				Tween 80	Tween 60	

Notes: Y1: EE (%), Y2: Q_12h_ (%), PS (nm), * the values are described as mean ± SD; all formulations involved 10 mg EGCG, *n* = 3, # optimized Formula. Abbreviations: EE, entrapment efficiency; Q_12h,_ % drug released after 12 h; PS, Particle Size; EA, edge activator.

**Table 2:** The output data of the 2^3^ factorial design of EGCG-loaded SNVs.

Responses	*R* ^2^	Adjusted *R*^2^	Predicted *R*^2^	Adequate Precision
EE% (Y1)	0.9502	0.9129	0.8009	14.698
Q_12h_(Y2)	0.9924	0.9867	0.9695	32.553
PS (Y3)	0.9618	0.9331	0.8470	15.996

Abbreviations: *R*^2^, R-squared or the coefficient of determination; Q_12h_, % EGCG released after 12 h; EE, entrapment efficiency of EGCG within the SNVs; PS, the particle size of EGCG-loaded SNVs.

**Table 3:** ANOVA for the 2^3^ factorial design of EGCG-loaded SNVs.

Independent Variable	Source	Sum of Squares	df	Mean Square	F-Value	*p*-Value
EE% (Y1)	Model	497.23	31	165.74	25.45	0.0046
	X1	92.66	1	92.66	14.23	0.0196
	X2	280.95	1	280.95	43.14	0.0028
	X3	123.62	1	123.62	18.98	0.0121
Q_12h_ (Y2)	Model	1022.56	3	340.85	173.49	0.0001
	X1	49.54	1	49.54	25.22	0.0074
	X2	898.93	1	898.93	457.55	<0.0001
	X3	74.09	1	74.09	37.71	0.0036
PS (Y3)	Model	43,375	3	14,458	33.30	0.0027
	X1	29,598	1	29,598	44.67	0.0012
	X2	3383.46	1	3383.5	14.89	0.0388
	X3	10,393	1	10,393	40.34	0.0080

Notes: Values of *p* < 0.05 investigate the significance of model terms, X1: Ratio of Span to EA, X2: Type of non-ionic surfactant, X3: Type of EA. Abbreviations: EE, entrapment efficiency; Q_12h,_ % drug released after 12 h; PS, particle size; df, degree of freedom.

**Table 4:** The calculated correlation coefficients for the in vitro release of EGCG and EGCG-loaded spanlastics employing different kinetic orders.

Formula	Zero Order	First Order	Higuchi Model	Hixson Crowell	Baker–Lonsdale
F1	0.9372	−0.9740	0.9751	0.9643	0.9867
F2	0.9123	−0.9517	0.9612	0.9398	0.9784
F3	0.9365	−0.9767	0.9730	0.9728	0.9793
F4	0.9073	−0.9595	0.9539	0.9460	0.9725
F5	0.9346	−0.9729	0.9765	0.9618	0.9809
F6	0.9493	−0.9777	0.9849	0.9697	0.9859
F7	0.9419	−0.9888	0.9782	0.9852	0.9920
F8	0.9643	−0.9944	0.9904	0.9912	0.9951
EGCG	0.9953	−0.9698	0.9998	0.9913	0.9887

**Table 5:** Ex vivo permeation parameters of F4, the conventional niosomes, and EGCG dispersion.

Formula	* J_ss_ (µg cm^−2^ h^−1^)	* K_P_ (cm h^−1^)	ER
EGCG dispersion	1.35 ± 0.11	0.0034 ± 0.12	-------
Niosomal formula	6.38 ± 1.23	0.0119 ± 0.14	4.73
F4	9.13 ± 1.34	0.0228 ± 0.13	6.76

Notes: * Each value represents average ± SD (*n* = 3), F4: the optimized EGCG-loaded SNVs. Abbreviations: J_ss_, the steady-state flux; K_P,_ the permeability coefficient; ER, Enhancement ratio.

**Table 6:** The calculated correlation coefficients for the ex vivo permeation of F4, the niosomal formula, and EGCG employing different kinetic orders.

Formula	Zero Order	First Order	Higuchi Model	Hixson Crowell	Baker–Lonsdale
F4	0.9779	−0.9954	0.9974	0.9911	0.9985
Niosomal formula	0.9642	−0.9820	0.9931	0.9768	0.9970
EGCG	0.8841	−0.8930	0.9396	0.8901	0.9263

**Table 7:** Determination of the elasticity of EGCG-loaded SNVs and the corresponding niosomes.

Formula	PS before Extrusion (nm)	PS after Extrusion(nm)	DI
F4	243.8 ± 1.24	240.9 ± 1.27	19.27 ± 1.2
Niosomes	287.4 ± 1.52	58.2 ± 1.14	1.48 ± 0.02

Notes: F4, the optimized EGCG-loaded SNVs; the values are presented as mean ± SD; *n* = 3. Abbreviations: DI, deformability index; PS, particle size.

**Table 8:** Effect of storage at 4–8 °C on the properties of the EGCG-loaded spanlastic formula (F4) and the corresponding niosomal formula.

Parameter	Spanlastic Formula		Niosomal Formula	
	Fresh	Stored	Fresh	Stored
Drug content (%)	98.22 ± 1.21	97.98 ± 1. 14	99.14 ± 0.82	90.22 ± 1.60
EE (%)	80.56 ± 1.57	78.88 ± 1.18	69.84 ± 1.20	60.22 ± 1.57
Q_12h_ (%)	84.98 ± 1.14	83.74 ± 0.88	97.11 ± 1.24	85.92 ± 1.37
% drug permeated	62.22 ±1.23	61.05 ± 1.33	50.22 ± 1.17	39.56 ± 1.22
DI	19.27 ±0.92	18.81 ± 0.72	1.48 ±0.12	0.69 ± 0.05

Notes: Each value is described as mean ± SD (*n* = 3); F4, the optimized EGCG-loaded spanlastics. Abbreviations: EE, entrapment efficiency; Q_12h_, % drug released after 12 h; DI, deformability index.

**Table 9:** Pharmacokinetic parameters of EGCG-loaded formulations.

Parameter	EGCG-Loaded SNVs	EGCG-Loaded Niosomes	EGCG Dispersion
C_max_ (µg ml^−1^)	0.58 ± 0.032	0.40 ± 0.025	0.19 ± 0.007
AUC_0–24_ (µg h ml^−1^)	6.54 ± 0.16	4.24 ± 0.12	2.34 ± 0.03
t_1/2_ (h)	17.43 ± 0.34	11.42 ± 0.21	3.7 ± 0.11
F_re_ %	279.48	181.20	--------

Abbreviation: C_max_: maximum blood concentration; AUC: area under the EGCG concentration-time curve; t_1/2_, elimination half-life; F_re_ %, % relative bioavailability.

## Data Availability

The data presented in the current study are available from the corresponding author upon reasonable request.

## Supplementary Materials

The following are available online at https://www.mdpi.com/1424-8247/14/1/68/s1, Table S1: The prescreening study for the formulation of EGCG-loaded SNVs using different rotation, Table S2: Prescreening study for selecting the most suitable levels of different independent variables required for preparing EGCG-loaded SNVs.
