# Supplementary material for: Formulation and Optimization of Nanospanlastics for Improving the Bioavailability of Green Tea Epigallocatechin Gallate
Source: Pharmaceuticals (Basel). 2021 Jan 15;14(1):68. doi: 10.3390/ph14010068 (PMC7831059; doi:10.3390/ph14010068)
Supplement: Supplementary file 1 [file pharmaceuticals-14-00068-s001.pdf]

Table S1. The prescreening study for the formulation of EGCG-loaded SNVs using different rotation speeds and sonication times.

Formula	Rotation speed (rpm)	Sonication time (min)	* EE%
P1	500	3	88.04±2.82
P2	500	5	64.11 ± 1.41
P3	1000	3	77.32±1.28
P4	1000	5	53.44 ± 1.36

Notes: * the values are described as mean ± SD (n = 3). Abbreviations: EE, entrapment efficiency.

Table S2. Prescreening study for selecting the most suitable levels of different independent variables required for preparing EGCG-loaded SNVs.

Formula	Non-ionic surfactant to EA ratio	Type of non-ionic surfactant	Type of EA	*EE%
**S1**	1:1	Cremophor RH	Brij 35	35.28±1.24
**S2**	1:1	Cremophor RH	Tween 80	50.32±1.78
**S3**	1:1	Cremophor RH	Tween 60	61.48±2.11
**S4**	1:1	Span 60	Brij 35	68.34±1.44
**S5**	1:1	Span 60	Tween 80	80.12±1.73
**S6**	1:1	Span 60	Tween 60	88.95±1.92
**S7**	1:1	Span 80	Brij 35	50.21±1.22
**S8**	1:1	Span 80	Tween 80	64.21±1.44
**S9**	1:1	Span 80	Tween 60	71.43±1.28
**S10**	3:2	Cremophor RH	Brij 35	44.60±1.34
**S11**	3:2	Cremophor RH	Tween 80	59.48±1.21
**S12**	3:2	Cremophor RH	Tween 60	67.34±1.95
**S13**	3:2	Span 60	Brij 35	74.59±1.33
**S14**	3:2	Span 60	Tween 80	88.04±2.82
**S15**	3:2	Span 60	Tween 60	92.35±1.34
**S16**	3:2	Span 80	Brij 35	57.11±1.48
**S17**	3:2	Span 80	Tween 80	70.53±1.81
**S18**	3:2	Span 80	Tween 60	80.56±1.57
**S19**	4:1	Cremophor RH	Brij 35	58.29±1.63
**S20**	4:1	Cremophor RH	Tween 80	70.33±1.28
**S21**	4:1	Cremophor RH	Tween 60	80.02±2.14
**S22**	4:1	Span 60	Brij 35	80.35±1.28
**S23**	4:1	Span 60	Tween 80	90.49±1.22
**S24**	4:1	Span 60	Tween 60	97.93±1.28
**S25**	4:1	Span 80	Brij 35	64.23±1.22
**S26**	4:1	Span 80	Tween 80	80.32±1.38
**S27**	4:1	Span 80	Tween 60	89.99±2.16

Notes: * the values are expressed as mean ± SD (n = 3). Abbreviations: EE, entrapment efficiency; EA, edge activator.
